# Swallowing performance during cognitive tasks in healthy adults

**DOI:** 10.3389/fneur.2026.1743003

**Published:** 2026-04-02

**Authors:** Xiuen Chen, Chenchen Zhang, Wenhui Shi, Zhichao Ning, Jiejiao Zheng, Yongjun Zheng, Yunyun Zhang

**Affiliations:** 1 Department of Rehabilitation, Huadong Hospital, Fudan University, Shanghai, China; 2 Department of Internal Neurology, Yueyang Hospital of Integrated Traditional Chinese and Western Medicine Affiliated to Shanghai University of Traditional Chinese Medicine, Shanghai, China; 3 School of Exercise and Health, Shanghai University of Sport, Shanghai, China; 4 Department of Rehabilitation Medicine, Shanghai Fourth Rehabilitation Hospital, Shanghai, China

**Keywords:** cognition, dual task, executive function, healthy adults, swallowing

## Abstract

**Objective:**

Dysphagia frequently co-occurs with cognitive impairment, suggesting that swallowing may involve cognitive processes. However, the relationship between cognitive load and swallowing performance remains unclear. This study aimed to examine the effects of different levels of cognitive load on swallowing performance under dual-task conditions in healthy adults.

**Methods:**

Forty-three graduate students (mean age: 24.49 ± 1.63 years, male/female: 22/21) were recruited and performed five tasks: swallowing single task (SST), low-load cognitive single task (ST_low_), high-load cognitive single task (ST_high_), low-load cognitive-swallowing dual task (DT_low_), and high-load cognitive-swallowing dual task (DT_high_). Here, a dual-task refers to the concurrent execution of a swallowing task and a cognitive task, designed to evaluate the competition for attentional resources and performance trade-offs. The low-load cognitive task consisted of a digit forward-span task, while the high-load cognitive task involved a digit backward-span task, which demands greater cognitive processing complexity and working memory load. Swallowing measures (e.g., swallowing volume) and cognitive task accuracy were recorded, and dual-task cost (DTC) was calculated to evaluate performance changes under dual-task conditions.

**Results:**

Swallowing volume differed significantly among SST, DT_low_, and DT_high_ (*p* < 0.05), with lower volume in DT_high_ compared to SST (*p* < 0.05). Cognitive accuracy was significantly lower in DT_high_ than in DT_low_ (*p* < 0.05), indicating greater interference under high cognitive load.

**Conclusion:**

The findings confirm that swallowing engages executive function and attentional resources, and suggest that participants prioritize swallowing performance during dual-task conditions by adjusting prefrontal activation patterns rather than increasing overall cortical activation to manage interference. This study provides preliminary evidence for the cognitive involvement in swallowing and highlights the need to consider cognitive load in dysphagia assessment and rehabilitation.

## 1 Introduction

Swallowing refers to the movement or process which a bolus formed by chewing was transported from the mouth to the stomach. Complex neuromuscular activities are involved to ensure the effectiveness and safety of swallowing (1). Dysphagia is caused by impaired organ structures or functions associated with swallowing that cannot safely and efficiently transport liquid bolus or liquids into the stomach. As a core symptom of most neurodegenerative diseases, dysphagia is more common in elderly patients diagnosed with neurological diseases such as Alzheimer's disease, Parkinson's disease, and stroke (2). In addition to aging and neurological disorders, cognitive decline is another independent risk factor for dysphagia (3). Studies have indicated that dysphagia is associated with an increased risk of aspiration pneumonia and malnutrition (4, 5), which in turn leads to more severe disability, increased length of stay and medical costs, and even death (6). Based on the above associations and the impact of cognitive and swallowing disorders on functional independence, the relationship between cognition and swallowing has received increasing attention. Some studies even suggest that there is no direct link between cognition and swallowing (7, 8), while others propose a certain degree of association (9). However, few studies have empirically investigated the connection between cognition and swallowing.

In daily life, swallowing is often carried out simultaneously with other activities, such as talking to others, watching TV or upper limb movement (10). Therefore, in order to test the influence of multitasking on swallowing and the potential participation of cognition, especially the allocation of attention resources, the “dual task paradigm” was mainly applied in the past experiments (11). Dual task (DT) refers to the execution of a parallel task while performing a motor task, which may further increase the attention demand and lead to a decline in the performance of one or both tasks compared to the performance of separate tasks (12). This phenomenon is called dual task interference. Dual task interference is mainly caused by two factors, one is the type of parallel task, the other is the complexity or load of parallel task (13). According to “capacity model theory” and “bottleneck theory”, performing two tasks with similar cognitive or motor demands will lead to a decline in the performance of one or both tasks; If the load of parallel task increases, more attention resources will be required, thus causing greater interference to the main task (14). Most of the previous swallowing dual-task studies only considered smaller swallowing test volumes, such as 5 ml and 10 ml, simulating single-oral swallowing. While, larger test volumes are better representative of continuous swallowing, making the study easier to generalize to real lifestyles. The cognitive task setting is relatively simple and does not even quantify cognitive performance, and some paradigms require response keys which results in inconsistent results of double-task interference in different studies. In addition, the effect of increased cognitive load on swallowing performance was not further explored (15–17). Therefore, optimizing the setting of swallowing task and parallel task, observing and comparing the influence of different types and loads of parallel tasks on swallowing will be helpful for us to further explore the influence of cognition on swallowing activity.

The purpose of this study was to observe and compare the performance of swallowing and parallel tasks in healthy adults performing different types and loads of swallowing dual tasks, so as to explore the relationship between swallowing and cognition, and provide more evidence for in-depth understanding of the brain network between the two.

## 2 Method

### 2.1 Participants

Forty-three participants were involved in this cross sectional study design. 22 males and 21 females within the age range of 18–26 years (mean age= 24.49 ± 1.63 years) participated. History of speech, language, hearing, neuro-logical deficits and/or history of surgery done to oro-pharyngeal apparatus were ruled out, ascertained by case history. Cognitive functioning was estimated to be intact as per Mini Mental Status Examination. Swallowing functioning was estimated to be normal by EAT-10. Right-handedness was confirmed by using the Edinburgh Dominant Hand Scale. Volunteer to participate in the study and sign the “informed consent.”

### 2.2 Procedures

Each participant was seated comfortably in an upright position on a chair. Swallowing ability was tested using thin liquids in the form of room-temperature water. Participants were instructed to take a 350-ml bottle of drinking water and swallow it within 16 s using a straw. To test the DT condition, cognitive tasks were incorporated, namely Digit span forward and Digit span backward tasks. Therefore, participants were required to perform five tasks, including swallowing single task, digit span forward task, digit span backward task, digit span forward-swallowing dual task and digit span backward-swallowing dual task. The participants will complete three single tasks and two double tasks in the random order generated by the random function on the Excel sheet. There will be a 5-min break between each task to prevent fatigue. The experimental tasks were designed as blocks, and E-prime software was used to complete the preparation, presentation, running and real-time recording of stimulus sequences on a 13.3 inch laptop screen. Participants could perform the task through the text prompts on the computer screen. The number of laryngeal elevations for each participant was measured from the video recordings. The video recordings of this study were specifically focused on the laryngeal area during the collection process, in order to ensure that the upward movement of the larynx could be clearly observed during the swallowing process. Before the formal measurement, each participant will practice the task and understand the experiment process. This research was conducted by two evaluators and two assistants. To mitigate emotional confounding—specifically, anxiety that arose from participants' uncertainty regarding task procedures or from physiological discomfort associated with excessive water intake—a senior clinician was responsible for baseline assessments and enrollment screening. This clinician also coordinated the experimental protocol to ensure participants fully understood the tasks and maintained a stable emotional state throughout the procedure. Another evaluator performed all task assessments for enrolled participants and recorded their behavioral performance. All video recordings were independently reviewed by two trained assistants who had completed a standardized training protocol using practice videos until reaching ≥90% pre-test consistency prior to formal data collection. The two assistants scored the recordings and jointly performed the subsequent statistical analysis.

#### 2.2.1 Baseline task (single task) assessment

**Figure 1:**
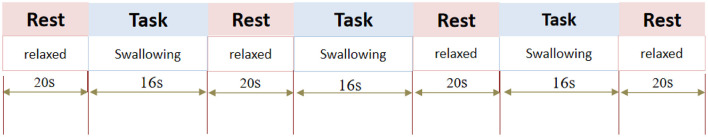
The imaging protocol and SST task arrangement.

**Figure 2:**
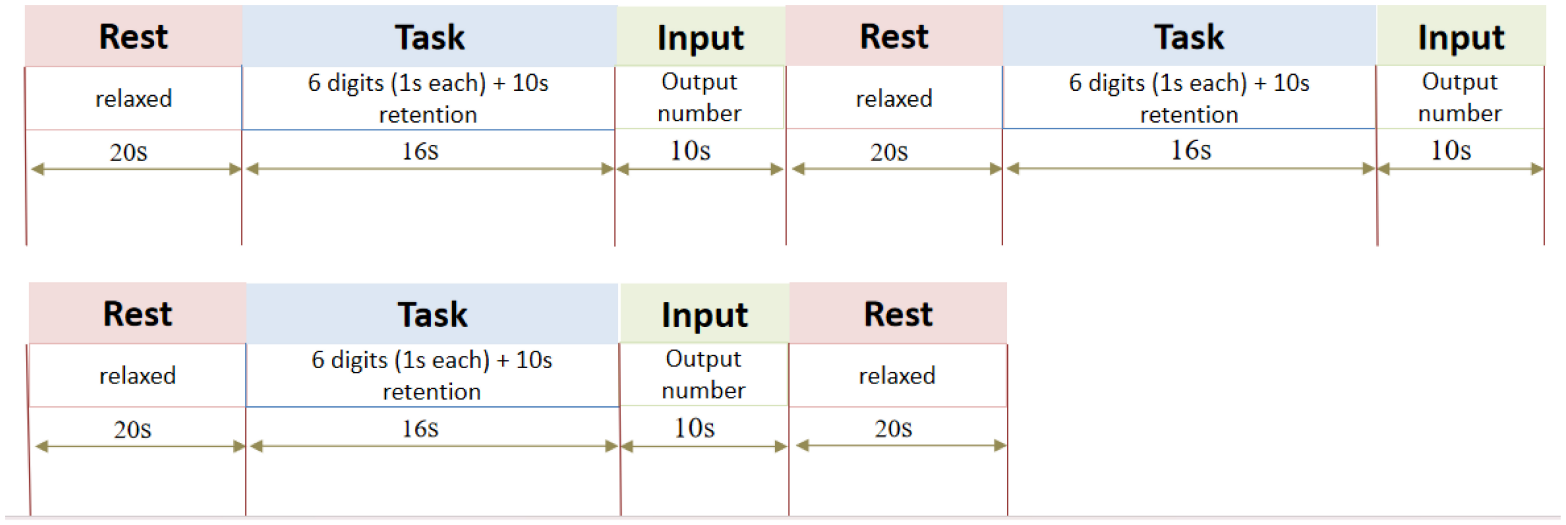
The chart shows three repeated cycles of the cognitive task sequence and ends with the last rest period. Each cycle includes “rest” (relaxed state, 20 seconds), “task” (each number is presented for 1 second, retained for 10 seconds, total 16 seconds), “input” (output of numbers, 10 seconds), and repeats. There is the same format between the three cycles.

① Swallowing single task: the swallowing task design consisted of 4 resting blocks and 3 task blocks, and the two blocks were presented alternately. The resting block lasted for 20 s, during which a black “+” symbol was displayed in the center of the screen, prompting the subject to remain relaxed. The task block was 16 s. Upon hearing the prompt sound, the subject swallowed water continuously through a straw at their habitual pace. The straw was connected to a 350 ml reservoir (*ad libitum* intake; no requirement to finish). Swallowing ceased immediately when the prompt sound ended. Each subject completed three 16 s blocks, with a fresh 350 ml bottle provided before each block. Swallowing frequency and volume (measured by bottle weight difference) were recorded per block as primary outcomes. The procedure is shown in Figure 1.
② Low-load cognitive single task (*ST*_*low*_) and High-load cognitive single task (*ST*_*high*_) : digit span forward task and digit span backward task were used, respectively. There were 3 task blocks and 4 resting blocks, and the two blocks were presented alternately. The resting block was 20 s, during which a black “+” symbol was displayed in the center of the screen to remind subjects to stay relaxed and avoid any intentional thinking activities. The task block was 16 s. Six random numbers ranging from 0 to 9 appeared successively on the screen, and each number was displayed for 1 s with no interval between them. After the number appeared, the screen was blank for 10 s, prompting subjects to organize and maintain memory. At the end of the 16 s task period, the prompt tone sounded again, and an input box appeared in the center of the screen, prompting the subjects to input the memorized number sequence through a numeric keypad, which was required to be completed within 10 s and kept relaxed again. The entire task flow is shown in Figure 2.

#### 2.2.2 Dual task assessment

**Figure 3:**
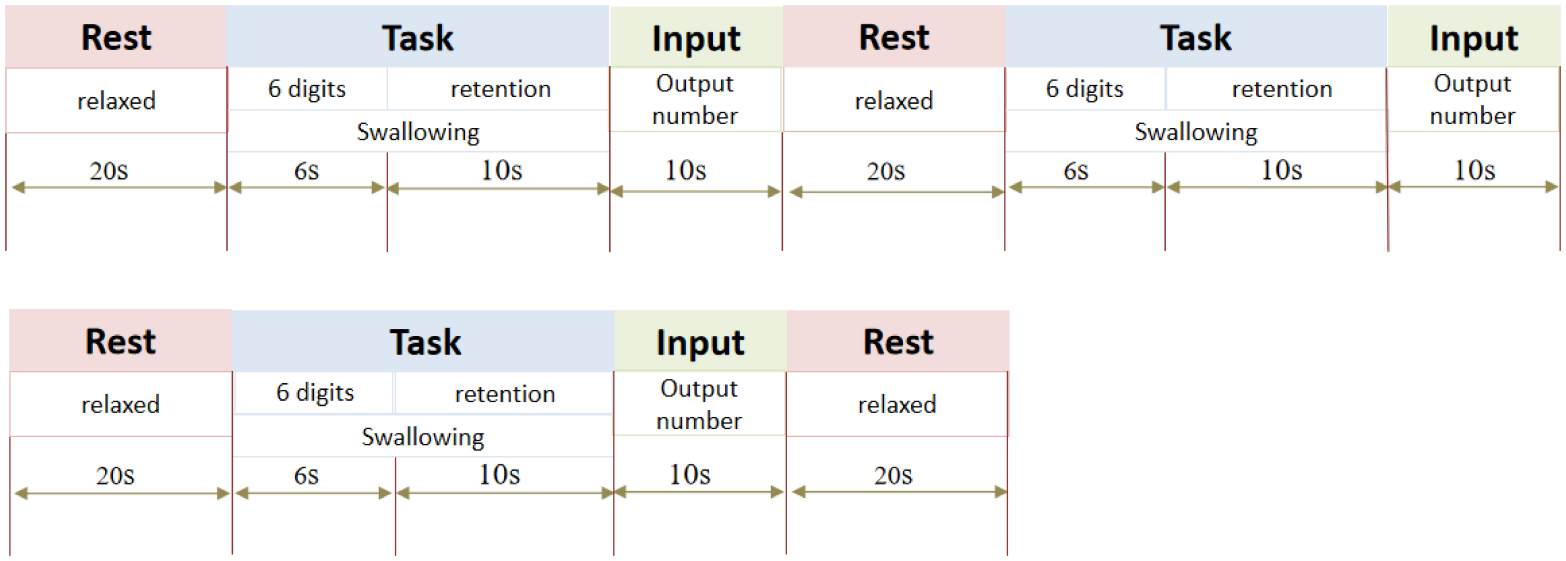
The imaging protocol and DT_low/high_ task arrangement.

① Low-load cognitive-swallowing dual task (*DT*_*low*_) and High-load cognitive-swallowing dual task (*DT*_*high*_): The parallel cognitive tasks are the digit span forward task and the digit span backward task, respectively, with 3 task blocks and 4 resting blocks, which are presented alternately. The resting block was 20 s, during which a black “+” symbol was displayed in the center of the screen to remind subjects to stay relaxed and avoid any intentional thinking activities. The task block was 16 s. The sound prompts the subjects to drink water continuously through a straw (one end of which is connected to a water bottle containing 350 ml drinking water). At the same time, 6 random numbers ranging from 0 to 9 appear successively on the screen, with each number showing 1 s and no interval between them. Prompt the subjects to organize and maintain memory. During this period, subjects were asked to pay close attention to both tasks simultaneously. At the end of the 16 s task period, swallowing should be stopped as soon as possible when the prompt sound sounded again. At this time, an input box appeared in the center of the screen, prompting the subjects to input the memorized number sequence through a numeric keypad, which was required to be completed within 10 s and kept relaxed again. Similarly, for each task block, the researchers replaced the water bottle in front of the subjects with another water bottle containing 350 ml of drinking water. The whole task process was shown in Figure 3, and the swallowing times and swallowing volumes of subjects in each block were recorded.

### 2.3 Swallowing and cognitive performance

① Swallowing performance: number of swallowing (NS), volume of swallowing (VS), volume per swallow (VPS), volume per time (VPT) and time per swallow (TPS) in each 16 s task block were recorded during SST, *DT*_*low*_ and *DT*_*high*_. Number of swallowing refer to numbers of the subject's laryngeal lifting within 16 s. Swallowing volume is the initial 350 ml in the cup at each task minus the amount of liquid remaining in the cup. Volume per swallow is swallowing volume/number of swallowing, volume per time is swallowing volume/16 s, and time per swallow is 16 s/swallowing times. The data measured in the three task blocks are averaged to get the mean value.
② Cognitive performance: the Eprime program recorded the accuracy of each output result of the subjects at *ST*_*low*_, *ST*_*high*_, *DT*_*low*_ and *DT*_*high*_, and the data measured in the three task blocks are averaged to get the mean value.
③ Dual costs: the effect of DT on task performance can be expressed by calculating the percentage change of related behavioral parameters between DT and ST, which is called dual task cost (DTC). In this study, the cost of accuracy (ACC) and volume of swallowing (VS) was calculated by the following formula:

Cognitive costs:


$$\begin{array}{l} {\text{ccDT}}_{\text{low}}=\frac{ACC\;DT_{low}\;-\;ACC\;ST_{low}}{ACC\;ST_{low}}\;\times 100\% \end{array}$$

$$\begin{array}{l} {\text{ccDT}}_{\text{high}}=\frac{ACC\;DT_{high}\;-\;ACC\;ST_{high}}{ACC\;ST_{high}}\;\times 100\% \end{array}$$

$$\begin{array}{l} {\text{ccDT}}_{\text{low}\to \text{high}}=\frac{ACC\;DT_{high}\;-\;ACC\;DT_{low}}{ACC\;DT_{low}}\;\times 100\% \end{array}$$

Swallowing costs:


$$\begin{array}{l} {\text{csDT}}_{\text{low}}=\frac{VS\;DT_{low}-\;VS\;ST_{swallow}}{VS\;ST_{swallow}}\;\times 100\% \end{array}$$

$$\begin{array}{l} {\text{csDT}}_{\text{high}}=\frac{VS\;DT_{high}-\;VS\;ST_{swallow}}{VS\;ST_{swallow}}\;\times 100\% \end{array}$$

$$\begin{array}{l} {\text{csDT}}_{\text{low}\to \text{high}}=\frac{VS\;DT_{high}-\;VS\;DT_{low}}{VS\;DT_{low}}\;\times 100\% \end{array}$$

### 2.4 Statistical analysis

Individuals have differences in their swallowing capabilities (such as the amount swallowed each time), which is an inherent characteristic of natural swallowing behavior. In this study, all participants underwent an assessment of their individual swallowing capabilities before the experiment, and the obtained scale results were compared. There were no significant differences between the baselines. The subsequent experimental results were statistically analyzed using the SPSS 25.0 software. Levene test was used for homogeneity of variance test, and Shapiro-Wilk test was used for normality test. Because the data were abnormal, the paired rank sum test was used to compare the data of two pairs, and the Friedman test was used to compare the data of multiple related samples, and the Bonferroni method was used for multiple comparison correction. The significance level was set at *P* < 0.05.

### 2.5 Ethics statement

The studies involving humans were approved by Hua-dong hospital Ethics committee (20220127). The studies were conducted in accordance with the local legislation and institutional requirements. The participants provided their written informed consent to participate in this study.

### 2.6 Clinical trial registration

https://www.chictr.org.cn/showproj.html?proj=187393.

## 3 Results

**Figure 4:**
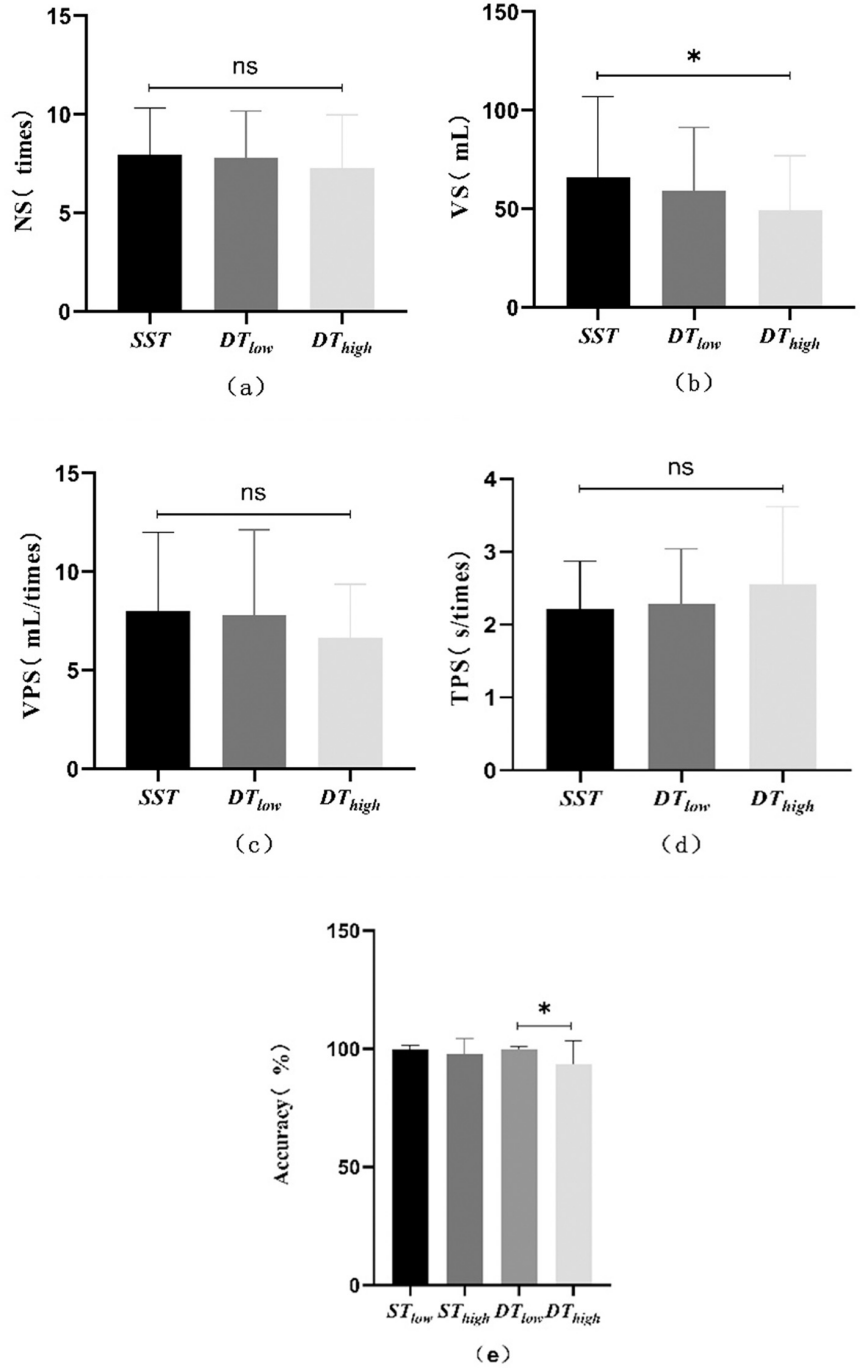
Swallowing and cognitive performances. **(a)** Comparison of the number of swallows (NS) across different tasks. **(b)** Comparison of the volume of swallow (VS) across different tasks. **(c)** Comparison of the volume per swallow (VPS) across different tasks. **(d)** Comparison of the time per swallow (TPS) across different tasks. **(e)** Comparison of accuracy across different tasks.

The demographic characteristics of the participants, swallowing/cognitive performance, and dual-task costs are presented in Tables 1–4 respectively. The swallowing and cognitive performance under different task conditions are further illustrated in Figures 4a–e.

**Table 1:** Demographic characteristics.

Gender (man/female)	Age (years)	Level of education (years)	Handedness (left/right)	MMSE (scores)	EAT-10 (scores)
22/21	24.49 ± 1.63	17.95 ± 0.78	0/43	30.00 ± 0.00	0.00 ± 0.00

**Table 2:** Swallowing parameters.

Wallowing measures	SST	DT_low_	DT_high_	*P*
NS (times)	7.96 ± 2.36	7.79 ± 2.38	7.30 ± 2.68	0.162
VS (ml)	66.06 ± 40.99	59.08 ± 32.41	49.17 ± 30.39	0.028
VPS (ml/times)	8.02 ± 3.97	7.78 ± 4.35	6.65 ± 2.72	0.124
TPS (s/times)	2.20 ± 0.66	2.29 ± 0.75	2.55 ± 1.07	0.184

**Table 3:** Cognitive parameters.

Cognitive measures	ST_low_	ST_high_	DT_low_	DT_high_	*P*
Accuracy (%)	99.73 ± 1.82	97.75 ± 6.61	99.71 ± 1.28	93.55 ± 9.93	0.000

**Table 4:** Swallowing and cognitive costs in percentage.

Dual-task cost measures		SST	DT_low_	DT_high_	ST_low_	ST_high_
Swallowing cost (VS)		SST → DT_low_ −0.16 ± 1.62%				
		SST → DT_high_ −15.56 ± 9.38^*^%				
			DT_low_ → DT_high_ −12.08 ± 5.29%			
Cognitive cost (ACC)	Cognitive load		DT_low_ → DT_high_ −6.14 ± 10.22^*^%		ST_low_ → ST_high_ −1.91 ± 7.03%	
	Swallowing		ST_low_ → DT_low_ 0.04 ± 2.46%			
				ST_high_ → DT_high_ −3.83 ± 12.21%		

### 3.1 Swallowing and cognitive performances

Volume of swallowing differed significantly among SST, DT_low_, and DT_high_ (Friedman test: *p* = 0.028), with a significant decrease in DT_high_ compared to SST (*post-hoc*: < 0.05). No significant difference in swallowing volume was found between DT_low_ and DT_high_ (*p* > 0.05).

Cognitive accuracy differed significantly among ST_low_, ST_high_, DT_low_, and DT_high_ (Friedman test: *p* = 0.000). The accuracy was significantly lower in DT_high_ compared to DT_low_ (*post-hoc*: *p* = 0.019, Figure 4e), but the decrease compared to *ST*_*high*_ was not significant (*p* > 0.05).

The cost of swallowing during dual-task was minimal under low cognitive load (csDT_low_, −0.16 ± 1.62%), while the cost of swallowing was significantly increased under high cognitive load (csDT_high_, −15.56 ± 9.38%, *p* < 0.05). The cognitive cost of DT_high_ (ccDT_high_, −3.83 ± 12.21%) was higher than that of DT_low_ (ccDT_low_, 0.04 ± 2.46%), but this difference was not significant (*p* > 0.05). In addition, compared with *DT*_*low*_, the increase of cognitive load led to a significant cognitive cost (ccDT_low_ → high, −6.14 ± 10.22%, *p* < 0.05), while the increase of swallowing cost was not significant (csDT_low_→_high_, −12.08 ± 5.29%, *P* > 0.05).

## 4 Discussion

The aim of this study is to investigate the relationship between swallowing and cognition by comparing the performance of swallowing and parallel tasks in healthy adults with different degrees of cognitive load. In previous studies, the parallel tasks of swallowing dual tasks mostly focused on one cognitive task or cognitive and motor tasks. This is the first study to examine the effects of dual-task swallowing with different levels of cognitive load on swallowing performance, cognitive performance. That study found that compared to SST, swallowing volume presented a significant decrease when performing DT_high_, while there was not significant swallowing cost during DT_low_. Compared to ST, the accuracy of cognitive tasks in DT condition was not affected by swallowing, but the accuracy of cognitive tasks in DT_high_ condition was significantly lower than that in DT_low_ condition.

The data from this study demonstrated that DT_high_ had significantly lower cognitive task accuracy than DT_low_, and swallowing volume was significantly reduced compared to SST, with significant swallowing costs and cognitive costs. The results have shown that digit span backward task (ST_high_) is more difficult than digit span forward task (ST_low_). In addition to encoding and silent maintenance of digit sequences, ST_high_ also includes digit sorting, which requires more brain activity and participation of a wider range of brain regions. Therefore, according to the capacity model theory, the greater dual-task costs observed under high cognitive load suggest that the concurrent performance of a demanding cognitive task and swallowing may compete for limited central resources. This interpretation is consistent with the view that swallowing, particularly its prehension stage, requires attentional resources beyond purely reflexive control. Previous studies of cognitive-gait dual tasks in the young and elderly (18) and stroke patients (19) have also found this significant interference which a significant reduction in gait speed in high-load cognitive-gait dual tasks compared with simpler cognitive demands. Researchers believe that this is due to the relatively complex cognitive components involved in high-load cognitive tasks and dual-task costs increase with the degree of interference between overlapping cognitive neural networks (20, 21). In previous studies on cognitive-swallowing dual tasks, healthy adults performed a number recognition task while swallowing 100 ml of liquid (16), a recognizing auditory stimuli task while swallowing 5 ml of liquid (15), and swallowing 300 ml barium while performing a series of cognitive-swallowing dual-task paradigms such as visuospatial orientation and repeated word repetition tasks (22), the decrease of swallowing efficiency has been observed. However, the 6-digit span forward task with relatively low cognitive load in this study did not cause significant effects on the simultaneous swallowing task, which is consistent with the results of Muhle et al. (17). Based on a synthesis of previous studies, we hypothesized that swallowing performance in young subjects varies with different loads of parallel tasks, and that this variability is negatively correlated with the degree of interference between overlapping neural networks. Previous research has characterized swallowing as a reflexive, vegetative, or automatic behavior driven by a central pattern generator (23). Therefore, it was thought that the neural circuits involved in swallowing were not involved in cognitive function controlled by cortical systems, or that attentional resources played little or no role in swallowing activity. According to this theory, DT should not have any effect on swallowing efficiency or cognitive performance. However, the “prehension stage” proposed by previous researchers, as well as the empirical study of dual-task swallowing in PD patients by Troche et al. (24), Brodsky et al. (25) and Reynolds et al. (26) help us to understand the participation and important role of cognitive components in swallowing activity. Therefore, under DT conditions, overlapping cognitive neural networks not only need to process the cognitive task, but also need to share their limited attention resources to perform the swallowing task, which will lead to dual-task interference. In general, the proposed “volume model” and “prehension stage” reveal an overlap in the neural network resources that control swallowing and cognitive activity, which goes beyond the “central pattern generator theory.”

Different from previous related studies, first of all, in terms of swallowing task volume setting, this study selected a large test volume to represent continuous swallowing which was close to the swallowing mode in real life, instead of single swallowing. Secondly, in terms of parallel cognitive tasks, this study different from the tasks that require finger tapping such as number recognition task and auditory stimulus response task, and the tasks that cannot quantify cognitive performance, such as repeated word silent reading and face recognition. This study selected the digital span paradigm that could adjust the difficulty load. Thirdly, in terms of task stimuli, this study adopted a block design which not only increased the compliance of the subjects, but also made the data more realistic and accurate by repeating swallowing movements and experimental trials.

## 5 Conclusion

In summary, when faced with swallowing dual tasks of varying cognitive load, subjects adjusted their behavior to meet the specific attentional demands of swallowing. The observed reduction in swallowing volume under high cognitive load, accompanied by a significant decline in cognitive task accuracy, could be interpreted in multiple ways. Alternative explanations such as general slowing, increased caution, or non-specific task interference might also account for some aspects of the performance changes. However, the pattern of results—specifically, the presence of significant swallowing cost only under high cognitive load, together with the significant cognitive cost when comparing DT_low_ to DT_high_–suggests a more strategic allocation of attentional resources. This study demonstrates that daily swallowing activities require the engagement of executive function and attention. Furthermore, the findings suggest the existence of a “swallowing priority” strategy during dual-task swallowing, wherein individuals implicitly prioritize airway protection when attentional resources become limited, even at the expense of concurrent cognitive performance.

## 6 Limitations and future directions

This study has several limitations. First, a low-load cognitive task (digit span forward) was selected to avoid manual responses, which were required in many previous swallowing dual-task paradigms. Given the overlapping motor cortical networks for swallowing and finger movements, this design minimizes motor-related confounds and enables a more specific assessment of cognitive-swallowing interference. However, it also limits generalizability to naturalistic distractions (e.g., conversation), where motor output is typically involved.

Second, the sequential swallowing paradigm used here reflects continuous drinking behavior, but does not represent all swallowing types (e.g., single-bolus command swallows). Whether the observed dual-task effects extend to discrete swallowing tasks warrants future investigation.

Third, a 16 s block design and a 350 mL water reservoir were adopted to standardize task duration and accommodate fNIRS block design, rather than to simulate *ad libitum* drinking. Although actual intake per block was far below 350 ml, the fixed time window remains a deviation from natural swallowing behavior. These trade-offs between internal validity and ecological relevance should be considered when interpreting the findings.

Fourth, swallowing frequency was measured by visual counting of laryngeal elevation, a method that is inherently subjective. Although two trained raters achieved high pre-test consistency (≥90%) and resolved discrepancies through consensus, no formal inter-rater reliability coefficient was calculated. Future studies should incorporate automated swallowing detection or videofluoroscopy to improve measurement objectivity.

Fifth, owing to anatomical differences, specifically the less prominent external laryngeal landmarks observed in young adult females relative to males, the visual assessment of laryngeal elevation may be comparatively more difficult in female participants. In the future, the swallowing surface electromyography technology will be applied to objectively record the swallowing performance during the task period, thereby obtaining objective and accurate data.

Sixth, the generalizability of our findings is limited by the sample characteristics, as all participants were young, healthy graduate students with high education levels. This population's high cognitive reserve may have reduced the dual-task interference effects observed in this study.

Finally, the clinical implications of our findings remain preliminary. The observed decline in swallowing volume under high cognitive load suggests that concurrent cognitive demands during dysphagia therapy may compete for attentional resources. Future research should directly examine whether reducing extraneous cognitive load facilitates the learning of behavioral swallowing techniques in patient populations.

## Data Availability

The raw data supporting the conclusions of this article will be made available by the authors, without undue reservation.
